# Georgia State Sanitarium

**Published:** 1899-03

**Authors:** 


### GEORGIA STATE SANITARIUM.

WE have just received the annual report of the Trustees of Georgia State Sanitarium for the fiscal year from September 1, 1897, to September 1, 1898. The report is thoroughly comprehensive and contains 135 pages of reading-matter. Few physicians, and certainly few of the laity, have any idea of the immensity of this institution. Situated at Milledgeville, the old capital city of the State, the institution is commonly known as the insane asylum. This latter word, however, grates upon one's sensibility and we are very glad to see the words "Georgia State Sanitarium" adopted, and trust that in the future physicians may speak of it as such. This change of name was brought about by an act of the legislature. There are six regular physicians connected with the institution who give their whole time to work in the institution.

The State of Georgia is fortunate in having such an able physician at the head of this institution as is Dr. T. O. Powell. His long service in this institution, coupled with his clear knowledge of nervous diseases makes him a man eminently qualified for the place.

The annual report of Superintendent Powell contains many facts which are not well known to physicians in general, and for that reason we take the liberty of making some quotations from that report.

On September 1st, 1897, there were white patients on hand, 1,515, and colored, 653; making a total of 2,168. There were received after that time 432 whites and 227 colored, making a total reception of 659.

There were discharged, removed, eloped and died during the year 501, leaving on hand September 1st, 1898, 2,326, of whom there are 43 whites and 9 colored on furlough not discharged.

The average number of patients under treatment during the year was 2,247, while the total number receiving treatment was 2,827.

It shows that the average number receiving treatment was 162 more than last year.

There are a great many interesting things contained in the report, and we only wish that we had space to enumerate them. Georgia should certainly be proud of this institution and support it handsomely.

